# Supplementary material for: Airway-resident T cells from unexposed individuals cross-recognize SARS-CoV-2
Source: Nat Immunol. 2022 Aug 29;23(9):1324–9. doi: 10.1038/s41590-022-01292-1 (PMC9477726; doi:10.1038/s41590-022-01292-1)
Supplement: Supplementary file 1 — Characteristics of BAL and PBMC donors. [file 41590_2022_1292_MOESM1_ESM.pdf]

nature portfolio

https://doi.org/10.1038/s41590-022-01292-1

Supplementary information

Airway-resident T cells from unexposed individuals cross-recognize SARS-CoV-2

In the format provided by the authors and unedited

Supplementary Table 1. Characteristics of BAL and PBMC donors

Gender	Age	Vaccine	Virus Spieces	Bronchoscopy (days post vaccine)	Bronchoscopy (days post challenge)
M	18	TIV	Rhinovirus	40	43
F	20	LAIV	Virus negative	40	43
M	44	N/A	Virus negative	75	N/A
F	20	TIV	Virus negative	114	117
M	18	LAIV	Virus negative	113	116
M	19	LAIV	Virus negative	127	130
M	18	LAIV	Virus negative	124	127
F	20	LAIV	Virus negative	120	123
F	19	LAIV	Virus negative	127	130
F	21	LAIV	Virus negative	133	136

M, male; F, female; TIV, tetravalent-inactivated influenza vaccine; LAIV, live attenuated influenza vaccine, N/A, non applicable.
