# Supplementary material for: Airway-resident T cells from unexposed individuals cross-recognize SARS-CoV-2
Source: Nat Immunol. 2022 Aug 29;23(9):1324–9. doi: 10.1038/s41590-022-01292-1 (PMC9477726; doi:10.1038/s41590-022-01292-1)
Supplement: Supplementary file 2 — Reporting Summary [file 41590_2022_1292_MOESM2_ESM.pdf]

nature portfolio

Corresponding author(s): Mala Maini, Daniela Ferreira

Last updated by author(s): Jun 23, 2022

Reporting Summary

Nature Portfolio wishes to improve the reproducibility of the work that we publish. This form provides structure for consistency and transparency in reporting. For further information on Nature Portfolio policies, see our Editorial Policies and the Editorial Policy Checklist.

Statistics

For all statistical analyses, confirm that the following items are present in the figure legend, table legend, main text, or Methods section.

n/a	Confirmed	
☐	☒	The exact sample size (*n*) for each experimental group/condition, given as a discrete number and unit of measurement
☐	☒	A statement on whether measurements were taken from distinct samples or whether the same sample was measured repeatedly
☐	☒	The statistical test(s) used AND whether they are one- or two-sided<br>*Only common tests should be described solely by name; describe more complex techniques in the Methods section.*
☒	☐	A description of all covariates tested
☐	☒	A description of any assumptions or corrections, such as tests of normality and adjustment for multiple comparisons
☐	☒	A full description of the statistical parameters including central tendency (e.g. means) or other basic estimates (e.g. regression coefficient) AND variation (e.g. standard deviation) or associated estimates of uncertainty (e.g. confidence intervals)
☐	☒	For null hypothesis testing, the test statistic (e.g. *F*, *t*, *r*) with confidence intervals, effect sizes, degrees of freedom and *P* value noted<br>*Give P values as exact values whenever suitable.*
☒	☐	For Bayesian analysis, information on the choice of priors and Markov chain Monte Carlo settings
☒	☐	For hierarchical and complex designs, identification of the appropriate level for tests and full reporting of outcomes
☒	☐	Estimates of effect sizes (e.g. Cohen's *d*, Pearson's *r*), indicating how they were calculated

Our web collection on statistics for biologists contains articles on many of the points above.

Software and code

Policy information about availability of computer code

Data collection	No software was used to collect data.
Data analysis	Software used for data/statistical analysis: FlowJo v.10.7.1; FACSDIVA v9.0; Prism 7.0; Excel v.16.16.09;

For manuscripts utilizing custom algorithms or software that are central to the research but not yet described in published literature, software must be made available to editors and reviewers. We strongly encourage code deposition in a community repository (e.g. GitHub). See the Nature Portfolio guidelines for submitting code & software for further information.

Data

Policy information about availability of data

All manuscripts must include a data availability statement. This statement should provide the following information, where applicable:

- Accession codes, unique identifiers, or web links for publicly available datasets
- A description of any restrictions on data availability
- For clinical datasets or third party data, please ensure that the statement adheres to our policy

All data analysed during this study are included in this published article and its supporting information files.

Human research participants

Policy information about studies involving human research participants and Sex and Gender in Research.

Reporting on sex and gender	Five female and five male donors participated in this study, detailed information is provided in Extended Data Table 1.
Population characteristics	Population characteristics and treatments have are described in Methods and Supplementary materials. Ten healthy, non-smoking, adults (aged 18 - 44 years), were enrolled in two different EHPC studies from 2016 to 2018. Participants were challenged intranasally with live Streptococcus pneumoniae (serotype 6B) and immunised with influenza vaccine (LAIV or TIV) where applicable. BAL samples were obtained through research bronchoscopy between 1 and 4 months post pneumococcal challenge.
Recruitment	All volunteers gave written informed consent. Adult (>18 years) were invited to participate via email and posters. Key eligibility criteria included capacity to give informed consent, no immunocompromised state or contact with susceptible individuals, no pneumococcal or influenza vaccine or infection in the last 2years and not having taken part in EHPC studies in the past 3years.
Ethics oversight	North West National Health Service Research Ethics Committee (Ethics Committee reference numbers: 14/NW/1460 and18/NW/0481, and Human Tissue Authority licensing number: 12548

Note that full information on the approval of the study protocol must also be provided in the manuscript.

Field-specific reporting

Please select the one below that is the best fit for your research. If you are not sure, read the appropriate sections before making your selection.

☒ Life sciences ☐ Behavioural & social sciences ☐ Ecological, evolutionary & environmental sciences

For a reference copy of the document with all sections, see nature.com/documents/nr-reporting-summary-flat.pdf

Life sciences study design

All studies must disclose on these points even when the disclosure is negative.

Sample size	Sample sizes are given for each figure throughout the paper when individual dots are not shown. Sample size can vary across figure panels depending on which stimulations were performed (limited by number of BAL cells recovered).
Data exclusions	No participant or individual samples were excluded after data was generated.
Replication	Due to limited sample availability experiments were not replicated.
Randomization	To fit the aim of the study, all samples were considered as from one group: individuals unexposed to Sars-CoV-2 (pre-pandemic). Experiments were performed with protocols optimised to reduce batch variation and to ensure mixing of experimental groups across batches e.g Flow cytometer parameters were consistent between runs (No MFI comparisons were performed, only gating and percentage of parent).
Blinding	Experiments were not randomized and the investigators were not blinded to allocation during experiments and outcome assessment since all samples belonged to a single untreated group. However, experimental set-up and controls ensured accurate analysis and interpretation.

Reporting for specific materials, systems and methods

We require information from authors about some types of materials, experimental systems and methods used in many studies. Here, indicate whether each material, system or method listed is relevant to your study. If you are not sure if a list item applies to your research, read the appropriate section before selecting a response.

Materials & experimental systems

n/a	Involved in the study	
☐	☒	Antibodies
☒	☐	Eukaryotic cell lines
☒	☐	Palaeontology and archaeology
☒	☐	Animals and other organisms
☐	☒	Clinical data
☒	☐	Dual use research of concern

Methods

n/a	Involved in the study	
☒	☐	ChIP-seq
☐	☒	Flow cytometry
☒	☐	MRI-based neuroimaging

Antibodies

Antibodies used	Detailed information regarding all antibodies used in this study are listed in the methods with manufacturer, clone, and dilution used. Antibodies used in this study: TNF FITC (BD bioscience, clone MAb11; 1:50), CD8α BV785 (Biolegend, clone RPA-T8; 1:100), IFN-g BV605 (BD biosciences, clone B27; 1:100), IFN-g APC (Biolegend, clone 4S.B3; 1:50), CD3 BUV805 (BD biosciences, clone UCHT1; 1:100), CD4 BUV395 or BV 421 (BD biosciences, clone SK3; 1:100), CD154 (CD40L) Pe-Cy7 (Biolegend, clone 24-31; 1:100), CD103 BV711 (Biolegend, clone ber-act8; 1:100), CD69 BV510 (Biolegend, clone fn50; 1:100), CD49a BUV395 (BD biosciences, clone SR84; 1:100), CD3 APC-H7 (Biolegend, clone SK7; 1:100), CD4 PerCP5.5 (Biolegend, clone SK3; 1:100), CD8 AF700 (Biolegend, clone SK1; 1:100), CD69 BV650 (Biolegend, clone FN5O; 1:100), CD103 BV605 (Biolegend, clone Ber-ACT8; 1:100), CD49a APC (Biolegend, clone TS2/7; 1:100), IFN-g PE (Biolegend, clone 4S.B3; 1:100), and TNF BV711 (Biolegend, clone MAb11; 1:100).
Validation	All antibodies were purchased from well established manufacturers and were validated by the vendor for species and target. e.g. BD biosciences, Biolegend in Knock-out/knock-in primary model systems to ensure biological accuracy in ISO 9001 certified facilities. Side-by-side lot comparisons are performed. Details of antibody clones have been included for cross-referencing of manufacturing company specification/validation processes. We further validated antibodies by titration to optimal concentrations and by using positive controls where possible (e.g. using populations known to express a certain marker or by polyclonal stimulation). Fluorescence minus one stains or unstimulated wells were used to define gates in Flowjo for all FACS assays. Negative controls were included in each run and positive controls where possible (PBMCs). All data is presented as background subtracted as described in the methods.

Clinical data

Policy information about clinical studies
All manuscripts should comply with the ICMJE guidelines for publication of clinical research and a completed CONSORT checklist must be included with all submissions.

Clinical trial registration	n/a
Study protocol	North West National Health Service Research Ethics Committee reference numbers: 14/NW/1460 and18/NW/0481. Human Tissue Authority licensing number: 12548.
Data collection	Data collection was conducted in the Liverpool School of Tropical Medicine (Liverpool, UK) from 2016 to 2018.
Outcomes	n/a

Flow Cytometry

Plots

Confirm that:

☒ The axis labels state the marker and fluorochrome used (e.g. CD4-FITC).

☒ The axis scales are clearly visible. Include numbers along axes only for bottom left plot of group (a 'group' is an analysis of identical markers).

☒ All plots are contour plots with outliers or pseudocolor plots.

☒ A numerical value for number of cells or percentage (with statistics) is provided.

Methodology

Sample preparation	Detailed sample preparation is given in methods. All FACS was performed on frozen and thawed PBMC or BAL isolated by density gradient separation. Peripheral blood mononuclear cells (PBMC) were isolated from heparinized blood samples using Pancoll (Pan Biotech) or Histopaque®-1077 Hybri-MaxTM (Sigma-Aldrich) density gradient centrifugation in SepMate tubes (StemCell) according to the manufacturer's specifications.
Instrument	BD biosciences LSRII and Fortessa-X20 flow cytometers.
Software	FACS DIVA version 9.0 was used on instrument and exporting .fcs files were analysed in FlowJo version 10.7.1 (TreeStar).
Cell population abundance	BAL was stimulated using non-adherent cells after 4 hours resting at 37oC. PBMC and BAL were stained and run without sorting or enrichment.
Gating strategy	Example gating strategy and plots are given in Extended Data Figure 1. Data is reported as a percentage of lymphocytes/ singlets/live/CD3+/CD4+ or CD8+ defining antigen specificity by production of IFNg, TNFa, CD40L or combinations of those.

☒ Tick this box to confirm that a figure exemplifying the gating strategy is provided in the Supplementary Information.
